# Diagnostic Challenges of Thyrotropin-Secreting Hypophyseal Macroadenoma Associated with Papillary Thyroid Carcinoma: Case Report and Literature Review

**DOI:** 10.3390/diagnostics15111313

**Published:** 2025-05-23

**Authors:** Juras Jocys, Romena Laukienė, Algirdas Edvardas Tamošiūnas

**Affiliations:** 1 Faculty of Medicine, Vilnius University, 03101 Vilnius, Lithuania; 2 Clinic of Internal Diseases and Family Medicine, Institute of Clinical Medicine, Faculty of Medicine, Vilnius University, Santariskiu 2, 08661 Vilnius, Lithuania; 3 Department of Radiology, Nuclear Medicine and Medical Physis, Institute of Biomedical Sciences, Faculty of Medicine, Vilnius University, Santariskiu 2, 08661 Vilnius, Lithuania

**Keywords:** thyrotropin-secreting pituitary adenoma, papillary thyroid carcinoma, secondary hyperthyroidism, resistance to thyroid hormone, transsphenoidal macroadenomectomy, suppressive levothyroxine therapy, total thyroidectomy

## Abstract

Background and Clinical Significance: The concurrent presence of a thyrotropin-secreting hypophyseal adenoma (TSHoma) with a thyroid malignancy, such as papillary thyroid carcinoma (PTC), is exceptionally rare and significantly complicates clinical diagnosis and management. This rare combination raises difficult decisions regarding the treatment sequence and carries the risk of exacerbating either or both conditions. Case report: We present the case of a 59-year-old female patient exhibiting persistent hyperthyroid symptoms with unusually normal TSH levels despite elevated thyroid hormone concentrations. Initial diagnostic imaging revealed a hypophyseal macroadenoma and a diffuse nodular goiter. After the macroadenoma diagnosis, the patient initially refused surgical intervention, and subsequent dopamine agonist therapy proved ineffective. Eight years later, during a routine follow-up, a thyroid ultrasound revealed a diffuse nodular goiter classified as EU-TIRADS 5, and papillary thyroid carcinoma was confirmed through fine needle aspiration biopsy. A total thyroidectomy and subsequent radioactive iodine therapy were performed. However, persistently elevated postoperative TSH levels remained despite high-dose levothyroxine therapy. Due to the increased risk of malignancy recurrence associated with elevated TSH levels, the patient consented to macroadenoma surgery. A successful transsphenoidal macroadenomectomy stabilized the patient’s condition, allowing for the normalization of TSH levels. Conclusions: This case underscores the importance of accurate differential diagnosis and highlights the challenges in managing TSH levels in patients with coexisting thyroid malignancies. With there being no clear guidelines for managing the combination of these conditions, decisions regarding treatment priority should consider the patient’s preferences, the risk of malignancy recurrence or progression, neurological symptoms, and the aggressiveness of the thyroid tumor.

## 1. Introduction

Thyrotropin-secreting pituitary adenomas are exceptionally rare functional pituitary tumors that cause secondary hyperthyroidism. These adenomas represent approximately 0.5–2% of all hypophyseal benign tumors [1]. The estimated prevalence is 1.0–2.8 per 1,000,000 individuals, with equal incidence among males and females [1,2,3,4]. The coexistence of thyrotropin-secreting pituitary adenomas with thyroid malignancies, such as papillary thyroid carcinoma (PTC), is extraordinarily rare. To our current knowledge, only 18 cases have been reported in the literature [5,6,7,8,9,10,11,12,13,14,15,16,17]. The overlap of these two diseases presents unique diagnostic and management challenges because the clinical features and laboratory findings of the two conditions may be very similar or exacerbate one another, complicating accurate diagnosis and treatment. It has been theorized that long-term continuous TSH stimulation of the thyroid gland may contribute to the development of thyroid malignancy [14,15].

## 2. Case Presentation

A 59-year-old Caucasian woman presented with classic symptoms of hyperthyroidism (tachycardia, heat intolerance, and excessive sweating). She had a medical history of nodular goiter that was established approximately 35 years ago. Thyroid ultrasound showed diffuse nodular thyroid goiter. Laboratory tests showed unusually normal TSH levels in comparison to elevated thyroid hormone levels: thyroid stimulating hormone (TSH): 3.1 mU/L, free thyroxine (FT4): 24.8 pmol/L, free triiodothyronine (FT3): 7.0 pmol/L and anti-thyroid peroxidase antibodies (ATPO): 0.6 kU/L. An initial attempt of treatment with thyreostatic medications (thiamazole 5 mg TID) was unsuccessful.

**Figure 1:**
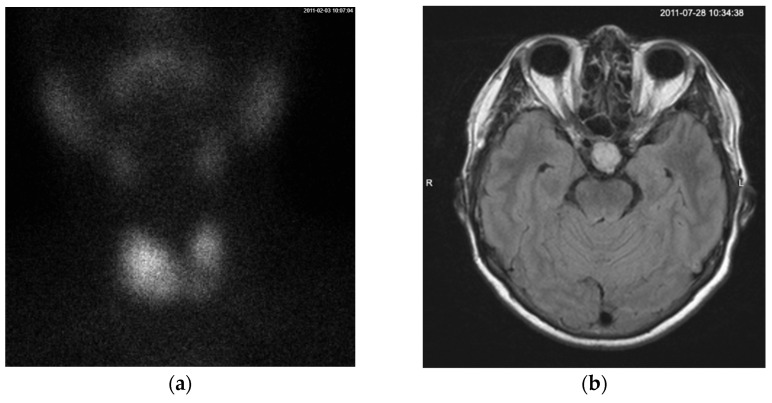
(**a**) On the Tc99 SPECT scan, the thyroid gland is in its typical anatomical position. The functional distribution is asymmetrical, with the left lobe smaller than the right lobe. Radiotracer uptake is observed on both sides, with more intense uptake in the lower two-thirds of the right lobe extending toward the isthmus. A pyramidal lobe uptake is visible superior to the isthmus. (**b**) MRI contrast enhanced T1-weighted axial scan showing a mass measuring 17 × 20 × 18 mm in the sella turcica region that is moderately enhanced by contrast material without a mass-compression effect on the surrounding structures, consistent with a macroadenoma. A cystic mass with well-defined borders in the right maxillary sinus was also noted.

A month later, the patient returned for follow-up examinations. Thyroid scintigraphy identified nodes of increased Tc99 uptake in both thyroid lobes, with more pronounced uptake in the inferior part of the right lobe (Figure 1a). A pituitary gland MRI confirmed the presence of a 17 × 20 × 18 mm macroadenoma with no neurological symptoms associated (Figure 1b). Same as during the consultation before, laboratory testing showed no additional hormonal abnormalities of the pituitary gland besides inadequately normal TSH level and slightly elevated sex hormone binding protein (SHBG) level at 103 nmol/L (laboratory reference value at the time being 50–80 nmol/L) (Table 1). The patient refused surgery, so treatment with dopamine agonists (bromocriptine) (the only state-funded medication for TSHoma treatment at the time) was attempted but proved ineffective.

**Figure 2:**
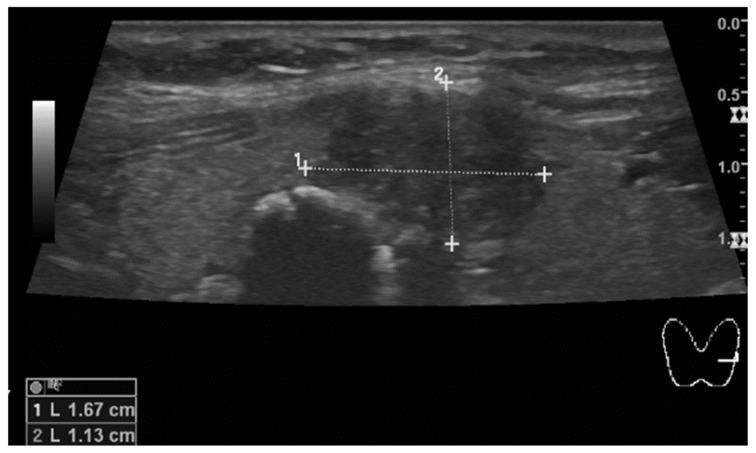
The hypoechoic node in the left thyroid lobe, close to the isthmus, measures 17 mm in diameter. A second smaller node with a prominent calcified wall and strong acoustic shadowing mimicking larix is also seen in the picture.

Eight years later, during a follow-up visit, thyroid ultrasound revealed a diffuse nodular goiter classified as EU-TIRADS 5 (Figure 2). A fine needle aspiration biopsy was performed, and the cytological analysis showed a Bethesda VI classification lesion, which strongly indicated the presence of papillary thyroid carcinoma. Total thyroidectomy with left central lymphadenectomy was performed (Table 2). The histological analysis revealed papillary carcinoma staged as pT2 pN1a. Metastases were found in two of six lymph nodes measuring 0.2 cm and 0.5 cm. Histopathological evaluation confirmed that the tumor was resected in full, with remaining non-cancerous thyroid nodular hyperplasia around.

Replacement levothyroxine therapy was started at a dose of 100 μg/day after surgery. However, TSH levels remained increased in spite of gradual increase in dose to 225 μg/day (Table 3). A higher dose of 250 μg/day was attempted; however, the patient showed signs of non-tolerance (tachycardia, hand tremor); therefore, she was treated with the maximum tolerated dose of levothyroxine 225 µg/day.

Postoperative thyroid scintigraphy I-131 showed increased uptake in postsurgical thyroid bed. Due to persistently elevated TSH levels and the risk of tumor recurrence, the patient underwent two cycles of I-131 therapy, after which the nodes of increased activity noted on the scintigraphy beforehand were no longer visible.

Control pituitary MRI examinations showed that the size of the macroadenoma remained the same, measuring 17 × 20 × 18 mm.

Despite the maximal tolerable dosage, the target TSH range of 0.1–0.5 mU/L was not achieved. Surgical treatment was recommended, but the patient refused the intervention. The inability to control TSH levels with the highest tolerable levothyroxine doses suggested possible thyroid hormone resistance syndrome (RTH). Genetic testing was performed, and no THRB and/or THRA genetic variants associated with RTH were found.

After one year, the patient agreed to transsphenoidal macroadenomectomy surgery. The surgery was successful, without any complications. The histopathological examination of the excised tissues showed the pituitary acidophilic stem cell PitNET/adenoma with Pit-1 and partial growth hormone (GH) and TSH expression. Other relevant findings were that somastatin receptor 2 (STTR2) expression was noted as three-thirds, somastatin receptor 5 (STTR5) expression was noted as one-third.

Postoperative MRI showed a deformed sella turcica region with a remaining heterogeneous mass measuring 11 × 16 × 11 mm moderately enhanced by contrast media, consistent with residual adenoma tissue. After surgery, the levothyroxine dose was reduced to 200 µg/day, resulting in a stable TSH level within the recommended range of 0.4–0.8 mU/L despite the remaining adenoma. Because postoperatively medical treatment with levothyroxine was successful, the patient does not report any adenoma-related symptoms and is scheduled for follow-up examinations one to two times per year. She was advised to repeat laboratory tests every six months and to undergo MRI and neck ultrasound examinations after one year.

## 3. Discussion

In cases presenting with TSHomas, a significant diagnostic challenge arises—one-third of these patients are initially misdiagnosed with primary hyperthyroidism [1]. As a result, they may undergo inappropriate treatments, such as thyroidectomy or Iodine-131 therapy, which can exacerbate the disease without addressing the existing pituitary tumor. When a TSHoma coexists with a thyroid malignancy, the situation becomes even more complicated, and decisions have to be made about which condition to treat first. This underscores the importance of considering secondary hyperthyroidism in the differential diagnosis for patients who present with elevated thyroid hormone levels and inappropriately normal or elevated TSH to avoid misdiagnosis and inadvertently worsening another coexisting disease, as illustrated in our case.

The usual clinical presentation for TSHomas includes classic thyrotoxicosis symptoms, such as heat intolerance, palpitations, weight loss, and diffuse goiter. The larger macroadenomas may also present with neurological symptoms (e.g., visual field defects, headaches) caused by compression of the anatomic structures surrounding the pituitary. Some TSHomas may cause symptoms due to an excess of other pituitary hormones (cases where the adenoma secretes not only TSH but other anterior pituitary hormones as well) [1,2,4].

In our case, the suspicion of secondary hyperthyroidism arose from typical thyrotoxicosis symptoms and abnormal laboratory test findings (normal TSH levels with elevated thyroid hormone levels), prompting us to perform a cerebral MRI, which revealed a mass in the pituitary gland. The presence of a pituitary lesion, combined with abnormal lab results and symptoms, strongly supports the diagnosis of a macroadenoma. However, it is important to note that further tests are necessary, since hypophyseal MRI findings can sometimes be incidentalomas that are not metabolically active. The main differential diagnosis of secondary hyperthyroidism lies between RTH (resistance to thyroid hormones) and TSHoma. Therefore, additional tests are needed to distinguish between the two [17].

From the 2013 guidelines of the European Thyroid Association on thyrotropinoma diagnostic recommendations, we were able to conduct most of the recommended laboratory tests with high to moderate quality of evidence, except for the TRH stimulation test, T3 suppression test, α-GSU (alpha-subunit of glycoprotein hormones), α-GSU/TSH ratio, and ICTP (type I collagen carboxy-terminal telopeptide) [18]. The TRH stimulation test has high-quality evidence for diagnosing TSHomas, showing abnormal TSH concentration responses in 90% of TSHoma patients—we could not perform this test because our hospital did not have the hormone in stock [18]. The T3 suppression test, which also has strong evidence for diagnosing TSHomas, has never been reported to show complete TSH inhibition in TSHoma patients, whereas partial TSH inhibition is usually seen in RTH. This makes it valuable for differential diagnosis, but it can pose risks for patients with increased cardiovascular risk, which was why we did not perform it in our case [18]. α-GSU and the α-GSU/TSH ratio have moderate-quality evidence and are used primarily for differential diagnosis. However, these tests were unavailable in our hospital and were, therefore, not performed. In patients with TSHoma, a high α-GSU/TSH ratio ([α-GSU (μg/L)/TSH (mU/L)] × 10) can be seen in approximately 80% of cases, and elevated α-GSU concentrations in about 70% of cases [18]. In contrast, patients with RTH typically have lower α-GSU/TSH ratios and lower α-GSU concentrations [17]. SHBG (sex hormone-binding globulin) and ICTP (type I collagen carboxy-terminal telopeptide) tests both have low-quality evidence and are not highly specific but they can still be somewhat useful for the differential diagnosis, as they are often elevated in cases of TSHoma [18]. The ICTP test was not available in our hospital; therefore, only SHBG levels were measured. Lastly, genetic testing, which has moderate-quality evidence, can be beneficial because most RTH cases are thought to result from genetic variants in the thyroid hormone receptor genes, THRB, and, less commonly, THRA [18,19,20]. We performed genetic testing for both THRB and THRA genes and found no genetic variants associated with RTH. Although helpful, the TRH stimulation test, α-GSU levels, and the α-GSU/TSH ratio are non-routine and often unnecessary for excluding RTH. In our case, RTH was ruled out based on negative genetic testing, the presence of a macroadenoma on MRI, and supporting laboratory findings, making TSHoma the most likely diagnosis.

The main reason why a thorough differential diagnosis is essential is that it determines the type of treatment the patient will undergo. Since 10–20% of pituitary adenomas are incidentalomas unrelated to functional TSH secretion, neurosurgery would be unnecessary and potentially harmful for these patients [17].

Eight years later, after the thyroid gland tumor was diagnosed, we were faced with a difficult decision on which condition to treat first. The first-line therapy for a TSHoma is surgical adenomectomy, typically performed transsphenoidally, although subfrontal approaches may be considered in certain situations [18]. If surgery is contraindicated or the patient refuses it, medical treatment with somatostatin analogs (lanreotide, octreotide) can be used [18]. Somatostatin analogs should be the first choice for medical therapy, as they normalize thyroid function in up to 90% of patients and can reduce adenoma size in up to 50% of cases [21,22,23,24]. Alternatively, treatment with dopamine agonists, such as cabergoline, is an option, though it has been shown to be less effective than somatostatin analogs; the best results with dopamine agonists are observed in “mixed” TSHomas that also secrete prolactin [23,24,25,26]. In our case, after the patient initially refused surgery, treatment with dopamine agonists was chosen over somatostatin analogs due to their coverage by state insurance, making them financially accessible for the patient, and thus the only viable option. However, the medical treatment with bromocriptine proved unsuccessful.

When aiming to normalize TSH levels, hypophyseal adenomectomy should be considered before total thyroidectomy to help slow tumor progression, reduce thyrotoxic symptoms, and decrease tumor vascularization, thereby better preparing the patient for thyroid surgery [10,12]. If the patient presents with neurological symptoms (e.g., visual field defects, headaches), this further supports performing an adenomectomy first [15]. It is also important to understand that performing a thyroidectomy first may accelerate macroadenoma growth due to the loss of negative feedback on the pituitary gland (while most TSHomas secrete hormones autonomously, some may still partially respond to negative feedback) [1,2]. This can lead to a rise in TSH levels and subsequent proliferation of any remaining thyroid tissue [2]. On the other hand, thyroidectomy may be prioritized over adenomectomy when the thyroid carcinoma is more aggressive and/or advanced compared to the TSHoma. In our case, the patient refused surgery for the macroadenoma, leaving us no choice but to proceed with thyroidectomy first.

TSH-suppressive hormonal therapy is a key component of postoperative management after total thyroidectomy for thyroid carcinoma with the aim of preventing neoplastic disease progression or relapse [7]. It becomes particularly challenging in those with coexisting TSHomas and thyroid malignancies, as in our case. Persistent elevation of TSH levels despite suppressive levothyroxine therapy is most often attributed to poor patient compliance, drug malabsorption, or increased metabolism induced by estrogen [12]. These causes can be ruled out by conducting a patient interview, monitoring adherence to treatment, performing repeated serum TSH measurements using different laboratory methods to eliminate potential inaccuracies, and assessing for biological interference with laboratory testing [17]. In cases of TSHomas, hormones are often secreted autonomously, disrupting the negative feedback loop and making it challenging to attain target TSH levels even with high suppressive levothyroxine doses, as seen in our case and multiple others [7,8,13,14,15,16,17]. Following thyroidectomy, the postoperative risk of tumor recurrence was classified as intermediate based on ATA guidelines (PTC with cervical lymph node metastases), leading to an initial target TSH level of 0.1–0.5 mIU/L [27]. We were unable to normalize TSH levels with a levothyroxine dose that was tolerable for the patient. When the patient was informed of the increased risk of thyroid malignancy relapse associated with uncontrolled TSH levels postoperatively, they agreed to the surgical removal of the macroadenoma. Once the patient underwent macroadenomectomy and additionally no clinical, biochemical, or structural evidence of thyroid malignancy remained, the initial recurrence risk was revised as recommended by the ATA guidelines. The patient was reclassified as having a low risk of recurrence with a target TSH concentration of 0.5–2 mIU/L [27].

Currently, there is no consensus or definitive guidelines on the most optimal treatment sequence for when these two conditions occur simultaneously. Each clinical case should be evaluated individually, taking into account the factors discussed previously along with the patient’s preferences, specific needs and other relevant considerations.

Lastly, it is important to consider that consistently higher than baseline TSH levels could potentially be a risk factor for the development of differentiated thyroid cancer due to continuous thyroid tissue stimulation. There are multiple studies that investigated the role of TSH in the development of PTC and carcinogenesis. One study showed a substantially higher incidence of papillary thyroid carcinoma in patients with higher levels of circulating TSH, another meta-analysis of 56 studies concluded a significant association between PTC and increased TSH levels [28,29,30].

## 4. Conclusions

This case contributes to the limited literature on the rare coexistence of TSHoma and papillary thyroid carcinoma. Diagnostic and management efforts are complicated by the limited availability of non-routine tests such as the TRH stimulation test, α-GSU, α-GSU/TSH ratio, and ICTP, also the inherent risks of some procedures, particularly the T3 suppression test which is contraindicated in elderly patients or in those with coronary heart disease. Another major challenge is controlling TSH levels after thyroidectomy, often requiring high-dose levothyroxine therapy, which usually proves inadequate without macroadenomectomy. Further studies are needed to determine the most effective treatment sequence for similar complex clinical scenarios.

## Figures and Tables

**Table 1:** Pertinent laboratory findings August 2011.

Test	Results	Normal Range
Thyroid stimulating hormone (TSH)	2.1 mU/L	0.4–4.0 mU/L
Free thyroxine (FT4)	24.7 pmol/L	9.0–19.0 pmol/L
Free triiodothyronine (LT3)	7.8 pmol/L	2.9–4.9 pmol/L
Prolactin (PRL)	285.6 mU/L	108.7–557.1 mU/L
Cortisol (COR)	404.0 nmol/L	101.0–536.0 nmol/L
IGF-1 (growth factor)	219.8 μg/L	43.0–220.0 μg/L
Sex hormone binding globulin (SHBG)	103.0 nmol/L	50.0–80.0 nmol/L

**Table 2:** Pre-operative laboratory findings.

Test	Results	Normal Range
Thyroid stimulating hormone (TSH)	2.1 mU/L	0.4–4.0 mU/L
Free thyroxine (FT4)	24.7 pmol/L	9.0–19.0 pmol/L
Free triiodothyronine (LT3)	7.8 pmol/L	2.9–4.9 pmol/L
Prolactin (PRL)	285.6 mU/L	108.7–557.1 mU/L
Insulin-like growth factor 1 (IGF-1)	121.0 µg/L	65–69 years: 40.2–225.0

**Table 3:** TSH changes dynamically after the thyroidectomy and after the I-131 therapy while gradually augmenting the hormone replacement therapy dosage.

Test and Biological Reference Values	August 2019 Post THR Surgery	September 2019	February 2020 Post I-131 Therapy	September 2020	April 2021	October 2021	May 2022	May 2022
TSH (0.4–4.0 mU/L)	9.3 ↑	8.2 ↑	5.0 ↑	3.7	2.8	2.0	2.4	1.3
Thyroglobulin (0.8–55.0 µg/L)	-	-	<0.2	<0.2	<0.2	<0.2	0.2	<0.2
Treatment (tab. Levothyroxine)	100 μg → 150 μg 1 t./d.	150 μg → 175 μg 1 t./d.	175 μg → 200 μg 1 t./d.	200 μg 1 t./d.	200 μg → 225 μg 1 t./d.	225 μg 1 t./d	225 μg 1 t./d	225 μg 1 t./d

## Data Availability

The original contributions presented in this study are included in the article. Further inquiries can be directed to the corresponding author.

## References

[1] Beck-Peccoz P., Giavoli C., Lania A. (2019). A 2019 update on TSH-secreting pituitary adenomas. J. Endocrinol. Investig..

[2] Beck-Peccoz P., Persani L., Mannavola D., Campi I. (2009). Pituitary tumours: TSH-secreting adenomas. Best Pract. Res. Clin. Endocrinol. Metab..

[3] Ónnestam L., Berinder K., Burman P., Dahlqvist P., Engström B.E., Wahlberg J., Nyström H.F. (2013). National incidence and prevalence of TSH-secreting pituitary adenomas in Sweden. J. Clin. Endocrinol. Metab..

[4] De Herdt C., Philipse E., De Block C. (2021). ENDOCRINE TUMOURS: Thyrotropin-secreting pituitary adenoma: A structured review of 535 adult cases. Eur. J. Endocrinol..

[5] Calle-Pascual A.L., Yuste E., Martin P., Aramendi T., Garcia-Mauriño M.L., Argente J., Catalan M.J., Uria J., Cabranes J.A., Charro A.L. (1991). Association of a thyrotropin-secreting pituitary adenoma and a thyroid follicular carcinoma. J. Endocrinol. Investig..

[6] Inoue H., Shinojima N., Ueda R., Yamamoto K., Ishii N., Igata M., Kawashima J., Araki E., Iwase H., Mikami Y. (2018). A Rare Case of Thyrotropin-Secreting Pituitary Adenoma Coexisting with Papillary Thyroid Carcinoma Presenting with Visual Disturbance without Hyperthyroidism. World Neurosurg..

[7] Gasparoni P., Rubello D., Persani L., Beck-Peccoz P. (1998). Unusual association between a thyrotropin-secreting pituitary adenoma and a papillary thyroid carcinoma. Thyroid.

[8] Yang J., Liu S., Yang Z., Shi Y.B. (2017). Ectopic thyrotropin secreting pituitary adenoma concomitant with papillary thyroid carcinoma: Case report. Medicine.

[9] Kishida M., Otsuka F., Kataoka H., Yokota K., Oishi T., Yamauchi T., Doihara H., Tamiya T., Mimura Y., Ogura T. (2000). Hyperthyroidism in a patient with TSH-producing pituitary adenoma coexisting with thyroid papillary adenocarcinoma. Endocr. J..

[10] Ohta S., Nishizawa S., Oki Y., Namba H. (2001). Coexistence of thyrotropin-producing pituitary adenoma with papillary adenocarcinoma of the thyroid—A case report and surgical strategy. Pituitary.

[11] Kiatpanabhikul P., Shuangshoti S., Chantra K., Navicharern P., Kingpetch K., Houngngam N., Snabboon T. (2017). A case of coexistence of TSH/GH-secreting pituitary tumor and papillary thyroid carcinoma: Challenges in pathogenesis and management. J. Clin. Neurosci..

[12] Gessl A., Vierhapper H., Feichtinger H. (2006). Non-suppressible TSH in a patient thyroidectomized due to follicular thyroid carcinoma. Exp. Clin. Endocrinol. Diabetes.

[13] Poggi M., Monti S., Pascucci C., Toscano V. (2009). A rare case of follicular thyroid carcinoma in a patient with thyrotropin-secreting pituitary adenoma. Am. J. Med. Sci..

[14] Ünlütürk U., Sriphrapradang C., Erdoğan M.F., Emral R., Güldiken S., Refetoff S., Güllü S. (2013). Management of differentiated thyroid cancer in the presence of resistance to thyroid hormone and TSH-secreting adenomas: A report of four cases and review of the literature. J. Clin. Endocrinol. Metab..

[15] Perticone F., Pigliaru F., Mariotti S., Deiana L., Furlani L., Mortini P., Losa M. (2015). Is the incidence of differentiated thyroid cancer increased in patients with thyrotropin-secreting adenomas? Report of three cases from a large consecutive series. Thyroid.

[16] Yoon J.H., Choi W., Park J.Y., Hong A.R., Kim S.S., Kim H.K., Kang H.-C. (2021). A challenging TSH/GH co-secreting pituitary adenoma with concomitant thyroid cancer; a case report and literature review. BMC Endocr. Disord..

[17] Safi S., Benabdelfedil Y., Derrou S., El Guendouz F. (2021). Simultaneous Coexistence of Thyrotropin-Prolactin-Secreting Adenoma and Papillary Thyroid Carcinoma. Case Rep. Endocrinol..

[18] Beck-Peccoz P., Lania A., Beckers A., Chatterjee K., Wemeau J.L. (2013). 2013 European thyroid association guidelines for the diagnosis and treatment of thyrotropin-secreting pituitary tumors. Eur. Thyroid. J..

[19] Beck-Peccoz P., Forloni F., Cortelazzi D., Persani L., Papandreou M.-J., Asteria C., Faglia G. (1992). Pituitary resistance to thyroid hormones. Horm. Res..

[20] Ortiga-Carvalho T.M., Sidhaye A.R., Wondisford F.E. (2014). Thyroid hormone receptors and resistance to thyroid hormone disorders. Nat. Rev. Endocrinol..

[21] Bertherat J., Brue T., Enjalbert A., Gunz G., Rasolonjanahary R., Warnet A., Jaquet P., Epelbaum J. (1992). Somatostatin receptors on thyrotropin-secreting pituitary adenomas: Comparison with the inhibitory effects of octreotide upon in vivo and in vitro hormonal secretions. J. Clin. Endocrinol. Metab..

[22] Socin H.V., Chanson P., Delemer B., Tabarin A., Rohmer V., Mockel J., Stevenaert A., Beckers A. (2003). The changing spectrum of TSH-secreting pituitary adenomas: Diagnosis and management in 43 patients. Eur. J. Endocrinol..

[23] Zgliczyński W., Zdunowski P., Czajka-Oraniec I., Jeske W., Zieliński G. (2013). The role of somatostatin analogues in treatment of TSH secreting pituitary adenomas. Thyroid. Res..

[24] Chanson P., Orgiazzi J., Derome P.J., Bression D., Jedynak C.P., Trouillas J., Legentil P., Racadot J., Peillon F. (1984). Paradoxical response of thyrotropin to L-dopa and presence of dopaminergic receptors in a thyrotropin-secreting pituitary adenoma. J. Clin. Endocrinol. Metab..

[25] Mouton F., Faivre-Defrance F., Cortet-Rudelli C., Assaker R., Soto-Ares G., Defoort-Dhellemmes S., Blond S., Wemeau J.-L., Vantyghem M.-C. (2008). TSH-secreting adenoma improved with cabergoline. Ann. Endocrinol..

[26] Mulinda J.R., Hasinski S., Rose L.I. (1999). Successful therapy for a mixed thyrotropin-and prolactin-secreting pituitary macroadenoma with cabergoline. Endocr. Pract..

[27] Haugen B.R., Alexander E.K., Bible K.C., Doherty G.M., Mandel S.J., Nikiforov Y.E., Pacini F., Randolph G.W., Sawka A.M., Schlumberger M. (2016). 2015 American Thyroid Association Management Guidelines for Adult Patients with Thyroid Nodules and Differentiated Thyroid Cancer: The American Thyroid Association Guidelines Task Force on Thyroid Nodules and Differentiated Thyroid Cancer. Thyroid.

[28] Dorange A., Triau S., Mucci-Hennekinne S., Bizon A., Laboureau-Soares S., Illouz F., Rodien P., Rohmer V. (2011). An elevated level of TSH might be predictive of differentiated thyroid cancer. Ann. Endocrinol..

[29] Kim H.K., Yoon J.H., Kim S.J., Cho J.S., Kweon S.S., Kang H.C. (2013). Higher TSH level is a risk factor for differentiated thyroid cancer. Clin. Endocrinol..

[30] Zheng J., Li C., Lu W., Wang C., Ai Z. (2016). Quantitative assessment of preoperative serum thyrotropin level and thyroid cancer. Oncotarget.

